# Decoding immune-related gene-signatures in colorectal neoplasia

**DOI:** 10.3389/fimmu.2024.1407995

**Published:** 2024-06-24

**Authors:** Thura Akrem Omran, Hege Smith Tunsjø, David Jahanlu, Stephan Andreas Brackmann, Vahid Bemanian, Per Christian Sæther

**Affiliations:** ^1^ Department of Life Sciences and Health, Oslo Metropolitan University, Oslo, Norway; ^2^ Division of Medicine, Department of Gastroenterology, Akershus University Hospital, Lørenskog, Norway; ^3^ Institute for Clinical Medicine, University of Oslo, Oslo, Norway; ^4^ Department of Pathology, Akershus University Hospital, Lørenskog, Norway; ^5^ Department of Immunology and Transfusion Medicine, Akershus University Hospital, Lørenskog, Norway

**Keywords:** colorectal cancer (CRC), immunology, biomarker potential, adenomatous polyps, gene expression, Norway

## Abstract

**Background:**

Colorectal cancer (CRC) is a significant health issue, with notable incidence rates in Norway. The immune response plays a dual role in CRC, offering both protective effects and promoting tumor growth. This research aims to provide a detailed screening of immune-related genes and identify specific genes in CRC and adenomatous polyps within the Norwegian population, potentially serving as detection biomarkers.

**Methods:**

The study involved 69 patients (228 biopsies) undergoing colonoscopy, divided into CRC, adenomatous polyps, and control groups. We examined the expression of 579 immune genes through nCounter analysis emphasizing differential expression in tumor versus adjacent non-tumorous tissue and performed quantitative reverse transcription polymerase chain reaction (RT-qPCR) across patient categories.

**Results:**

Key findings include the elevated expression of CXCL1, CXCL2, IL1B, IL6, CXCL8 (IL8), PTGS2, and SPP1 in CRC tissues. Additionally, CXCL1, CXCL2, IL6, CXCL8, and PTGS2 showed significant expression changes in adenomatous polyps, suggesting their early involvement in carcinogenesis.

**Conclusions:**

This study uncovers a distinctive immunological signature in colorectal neoplasia among Norwegians, highlighting CXCL1, CXCL2, IL1B, IL6, CXCL8, PTGS2, and SPP1 as potential CRC biomarkers. These findings warrant further research to confirm their role and explore their utility in non-invasive screening strategies.

## 1 Introduction

Colorectal cancer (CRC) is one of the most common cancer types and the leading cause of cancer-related mortality and morbidity across the world. Norway has one of the world’s highest rates of CRC, with 4745 newly diagnosed cases in 2022 (1). The reasons for such a high incidence of CRC in Norway remain unknown. Environmental factors such as diet and lifestyle, as well as genetic disposition, contribute to the development of CRC (2). Epigenetic changes and genetic abnormalities mark the development of CRC, transforming normal epithelial tissues into aggressive carcinomas (3). The somatic mutations in the Adenomatosis Polyposis Coli (APC) gene stand out for their critical role in initiating and driving the Wnt signaling pathway, marking an essential early event in the pathway to cellular transformation (4, 5).

The immune system has a significant impact on the development of CRC due to its intricate network of cells and molecules. One perspective is that it functions as a protector, identifying and eliminating cancer cells through diverse immune reactions (6). However, the immune system can also support the growth and advancement of tumors. Immune cells in the tumor microenvironment (TME) can be utilized by cancer cells to establish an immunosuppressive environment, which helps the tumor evade immune detection (7). This phenomenon is especially noticeable in CRC, where the interaction between various immune cells and cancer cells can have a significant impact on the progression of the disease and the effectiveness of treatment (8).

Comprehending CRC immunology requires understanding the intricate interactions within the tumor microenvironment (TME), including immunological checkpoints, cytokines, and both pro- and anti-inflammatory factors (9). This understanding is essential for predicting the course of disease progression and patient prognosis (10). Tumor-associated fibroblasts, vascular cells, and infiltrating immune cells interact with transformed cells in a reciprocal manner and generate an inflammatory milieu that may restrict or promote the growth of tumor cells (11). Studies have demonstrated that the presence of type 1 helper T cells (Th1) and cytotoxic lymphocytes indicates a more favorable outcome (8). Furthermore, CRC tumors characterized by microsatellite instability and mismatch repair deficiency exhibit increased infiltration of cytotoxic lymphocytes and demonstrate enhanced responsiveness to immune checkpoint blockade (12, 13). This highly immunogenic response is often associated with elevated levels of interferon-gamma (IFN-γ). In contrast, many other types of inflammation promote tumor growth. For instance, pro-inflammatory cytokines like interleukine-1 (IL-1), tumor necrosis factor alpha (TNF-α), and interleukin-6 (IL-6) may act directly on transformed cells to induce *de novo* transcription and promote cell survival (14, 15). Transforming Growth Factor Beta (TGF-β) produced by tumor cells suppresses immunogenic responses, and reactive oxygen species produced by myeloid-derived cells recruited to the TME may induce mutations in tumor cells (16).

Cytokines produced in the cancer’s TME may disseminate throughout the circulatory system; cytokine levels correlate with cancer presence, severity, and treatment efficacy. This makes them potential biomarkers for diagnosis and prognosis (17, 18). Researchers have studied various biomarkers, such as stool-based markers and blood-based assays, to determine their clinical relevance in the early detection of cancer (19, 20), and several studies have demonstrated the potential of cytokines and chemokines as non-invasive biomarkers (21, 22). In an early study by Krzystek-Korpacka et al., significant variations in circulating cytokine levels were observed between individuals with CRC, those at high risk for the disease, and healthy individuals. Cytokines like Interleukin 1 Beta (IL-1β), IL-6, C-X-C Motif Chemokine Ligand 8 (CXCL8 or IL-8), and Tumor Necrosis Factor Alpha (TNF-α) were found to be higher in individuals with advanced CRC (23). Czajka-Francruz et al. reviewed the interleukin profiles of patients with early and advanced CRC and provided clinical data on cytokines secreted by various cells in the TME, including differentiated T cells (24). Despite the promising outcomes of these studies, they only investigated the expression of a limited number of cytokines. Larger panels of immune genes could reveal more information about their roles in tumor progression and potentially identify new biomarker candidates. Creating a solid foundation for biomarker research is essential in cancer studies (25). While exploring immune profiles in tumors and nearby non-cancerous tissues has identified many potential biomarkers, inconsistencies across studies highlight the necessity for further research to confirm their reliability for clinical use (26). Additionally, limited comprehensive studies have been conducted on Norwegian CRC patients (27, 28). Cytokine and chemokine profiles may exhibit substantial variation due to genetic, environmental, and lifestyle factors specific to different populations (29, 30).

Furthermore, the expression of immune-related genes in adenomatous polyps has received little attention. Studying the immune response in adenomatous polyps could improve our understanding of the relationship between immune gene expression and early cancer development. This research may also identify biomarkers that differentiate adenomas from cancerous lesions (31). However, the immunological context of pre-cancerous colorectal polyps has not been studied as extensively as colorectal cancer (32). Several studies have shown that, similar to CRCs, colorectal adenomas also display the presence of interleukin 10 (IL-10)-producing regulatory T cells and CXCL8 overexpression (33–35).

Our study contributes to a detailed screening of immune-related genes in cancer and adjacent non-neoplastic tissues, with further investigation at multiple colonic sites in cancer, adenomatous polyps, and healthy controls. This study aims to provide extended insight into immunological signatures in colorectal tumors and identify immune-related genes that may differentiate cancer and adenoma patients in the Norwegian population.

## 2 Materials and methods

### 2.1 Study cohort and sample collection

The study cohort included 69 patients who were scheduled for colonoscopy at Akershus University Hospital (Ahus) between 2014 and 2017. Colonoscopy examinations were performed for a variety of reasons, including gastrointestinal bleeding, weight loss, changes in bowel habits, or the discovery of polyps or malignancies on computed tomography (CT) colonography. Participants with a history of inflammatory bowel disease were excluded from the study. All samples were collected during the initial colonoscopy, prior to the diagnosis. Biopsy samples were obtained before the initiation of any CRC treatment. The participants were categorized into three groups according to the results of the colonoscopy: patients with CRC (n = 25), patients with adenomatous polyps ≥ 10 mm (n = 25), and patients with no abnormalities seen during the colonoscopy, defined as controls (n = 19). Colonic mucosal biopsies measuring 2–3 mm were taken from individuals diagnosed with cancer and adenomatous polyps at four key locations: the ascending colon (AC), the cancerous tissue or polyp (TU), the non-neoplastic tissue adjacent to the cancer or polyp (located 5 cm away) (NN), and the sigmoid colon (CS). For comparison, samples were also collected from the ascending and sigmoid colons of the control individuals. Detailed patient and sample information is included in **Table 1**; **Supplementary Table 3**. The biopsies were preserved in Allprotect Tissue Reagent (Qiagen, Hilden, Germany) following the recommendations provided by the manufacturer.

**Table 1:** Characteristics of the study group.

	Cancer (n=25)	Polyp (n=25)	Control (n=19)	Total
**Age, mean (SD)**	69.3	66.9	58.5	NA*
**Female, n (%)**	7 (26.9%)	11 (42.3%)	8 (30.8%)	26
**Male, n (%)**	18 (41.8%)	14 (32.6%)	11 (25.6%)	43
**Total**	25 (36.2%)	25 (36.2%)	19 (27.6%)	69
Location: n (%)				
**Cecum**	6 (24%)	5 (20%)	NA	11
**Ascending colon**	3 (12%)	1 (4%)	NA	4
**Transverse colon**	2 (8%)	5 (20%)	NA	7
**Sigmoid colon**	11 (44%)	10 (40%)	NA	21
**Rectum**	3 (12%)	1 (4%)	NA	4
**Unknown**	0	3 (12%)	NA	3

*NA, Not available. Demographic and clinical characteristics.

### 2.2 Study design

**Figure 1:**
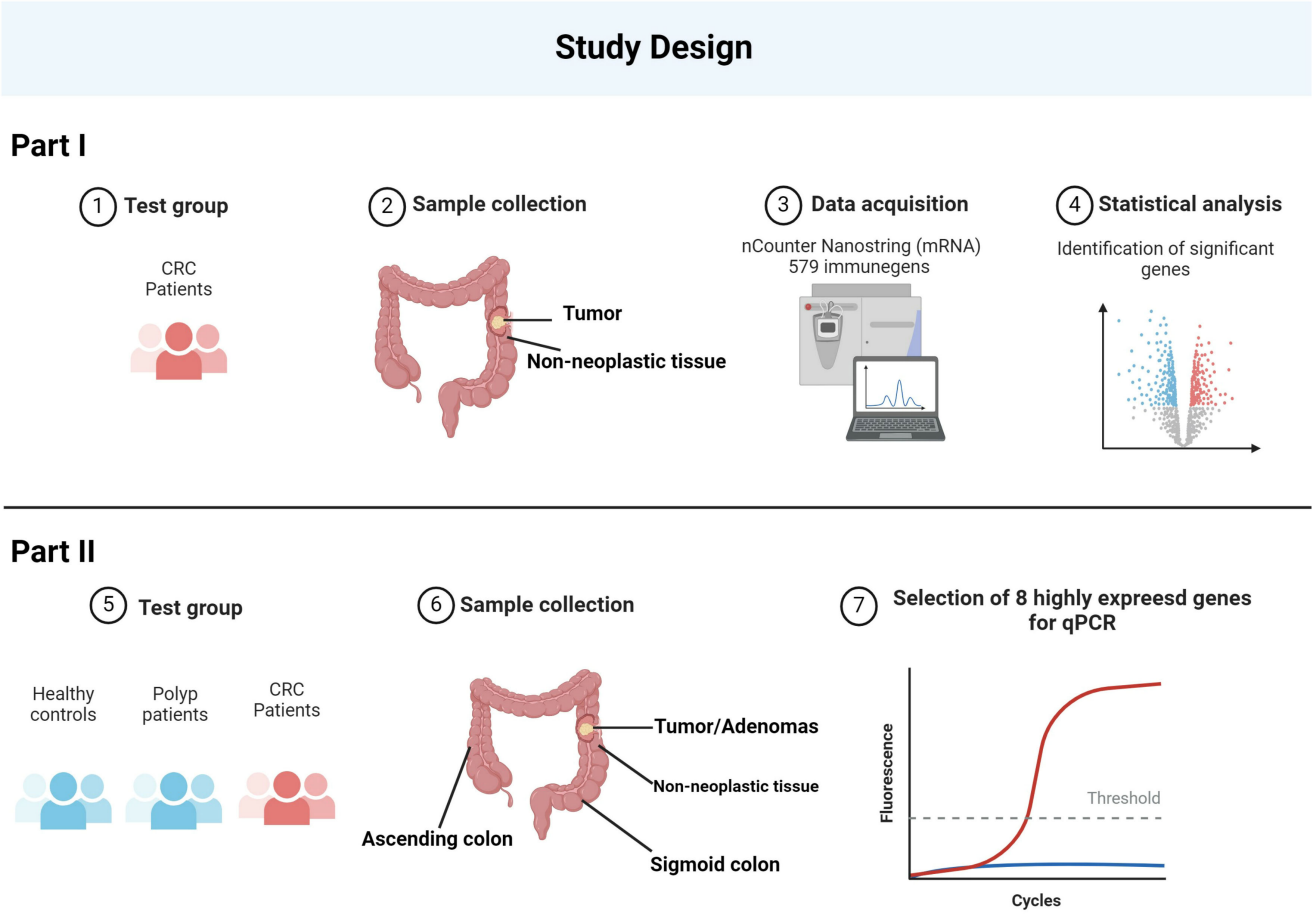
The schematic represents the study design. The study began with the collection of samples from CRC patients, including both tumor and adjacent non-neoplastic tissue. Utilizing the nCounter NanoString technology, the expression levels of 579 immune-related genes were quantitatively measured. From this comprehensive analysis, eight genes were identified as being most highly expressed. To validate these findings, we expanded the study to include healthy controls, polyp patients, and CRC patients, taking samples from various regions of the colon. The eight identified genes underwent further RT-qPCR analysis across these groups, confirming their elevated expression in CRC. Created with BioRender.com.

Initially, the expression of 579 immune genes was compared between tumor and adjacent non-neoplastic tissue in cancer patients using the nCounter technology (NanoString Technologies, WA, USA). Immune genes that were highly expressed in the majority of tumor samples were then selected for further analysis. A total of eight target genes were analyzed using reverse transcription quantitative polymerase chain reaction (RT-qPCR) on the complete sample set consisting of biopsies from four locations in cancer patients and patients with adenomatous polyps and from two locations in healthy controls (**Figure 1**).

### 2.3 Nucleic acid extraction

Nucleic acid RNA extraction was performed based on Moen et al. (36), modified from the AllPrep DNA/RNA mini kit (Qiagen). The modifications improved lysis and homogenization using mechanical (silica beads), chemical (lysis buffer), and enzymatic methods. Nucleic acids were stored at -80°C until further analysis. Total RNA quantity was measured using a Qubit 3.0 spectrophotometer with a Qubit RNA HS assay kit (Life Technologies, CA, USA). Nucleic acid purity was assessed on a Nanodrop 2000 spectrophotometer (Thermo Fisher Scientific, Waltham, MA, USA) by calculating A260/280 and A260/230 ratios.

### 2.4 Gene expression using the nCounter Human Immunology v2 panel

The nCounter Human Immunology v2 panel (NanoString Technologies) includes 579 genes involved in immunity and inflammation, along with 15 housekeeping genes. The technology uses solution-based mRNA hybridization to streptavidin-conjugated short probes glued to a biotin cartridge. All steps were performed according to the manufacturer’s instructions. Briefly, to hybridize, 5 µL of sample (25–24ng/µl) was mixed with hybridization buffer and probes (reporter and capture) and incubated at 65°C for 24 hours. The nCounter prep station processed all samples. The nCounter Digital Analyzer scanned cartridges to create reporter code counts (RCC) to quantify gene expression.

### 2.5 Data normalization

For data quality evaluation, background thresholding, and normalization, the raw data was processed using nSolver 4.0 software (NanoString Technologies). The background level was calculated by adding the two standard deviations (SD) to the mean counts of eight negative control probes. Samples failing to meet criteria such as the presence of detection or control linearity flags and recognition of fewer than 50% of probes above background were excluded. Using the geometric mean of housekeeping genes, the raw data was first normalized. Based on the established criteria, which required a normalization factor below three and a mean square error under 0.5 for the housekeeping gene to ensure sufficient RNA quality, none of the samples were excluded from the study. To assess the repeatability of the assay, one patient sample was used as a control and analyzed in each panel with a new lot number. GraphPad Prism 9 software (GraphPad Software, Inc., La Jolla, USA) was used to quantify the intensity and direction of this relationship through a linear regression analysis. The R^2^ for linear regression was 0.9983. NanoString Technologies considers R2 > 0.95 to be the gold standard for repeatability (37).

### 2.6 Differentially expressed genes (DEGs)

Heatmap analysis was done using Rosalind for Nanostring (Rosalind, San Diego, USA) to examine gene expression trends across samples. Using partitioning around medoids, differentially expressed gene heatmap clustering was completed. This was done using fpc R2. The heatmap was created using the samples’ 579 immune genes’ normalized gene expression levels. We performed a Wilcoxon-signed rank test to compare the expression differences of immune genes within CRC tumors and adjacent non-neoplastic tissue. A two-stage step-up method (Benjamini, Krieger, and Yekutieli) was used to conduct a false discovery rate (FDR) analysis at a 5% significance level. The Q value is used as a measure of the minimum FDR at which a test may be called significant. “q-value” is often used to refer to the adjusted p-value. All the statistical analyses were conducted using SPSS 27.0 software (IBM, IL, Chicago) and GraphPad Prism 9 (GraphPad Software, Inc.).

### 2.7 Gene expression analysis by real-time RT-PCR

Based on results from nCounter, RT-qPCR analyses targeting a selection of genes were conducted on all samples from the three patient groups: in total, 93 samples from cancer patients, 98 samples from polyp patients, and 37 samples from the control group. The analyses were performed using Applied Biosystems’ pre-built TaqMan assays and SuperScript™ III One-Step RT-PCR System with Platinum™ Taq DNA Polymerase (Invitrogen, ThermoFisher Scientific) using 10 ng total RNA in each reaction (**Supplementary Table 2**). The QuantStudio5 real-time PCR system (ThermoFisher Scientific) was used for RT-qPCR analysis with the following protocol: reverse transcription at 50°C for 20 minutes, Taq DNA polymerase activation at 94°C for 2 minutes, and 40 PCR cycles with denaturation at 94°C for 15 seconds and annealing and extension at 60°C for 30 seconds. For accuracy, three of the PCR assays [IL6, CXCL8, and CXCL1 (C-X-C Motif Chemokine Ligand 1)] were analyzed in technical duplicates using all samples (n = 228, in total 1368 PCR reactions). Wilcoxon’s test confirmed no difference between the duplicates; therefore, the remaining PCR assays were performed as single reactions. Amplification efficiencies were evaluated with LinRegPCR and used for correction of all qPCR data (38). To account for variations in sample quantity, normalization with reference genes was performed. For the identification of stably expressed reference genes, the transcription stability of four reference genes was investigated using NormFinder and BestKeeper (39, 40). Glyceraldehyde 3-Phosphate Dehydrogenase (GAPDH) and Polymerase (RNA) II (DNA-Directed) Polypeptide A (POLR2A) were evaluated as the best pair of reference genes (**Supplementary Table 2**). Transcription profiles were compared using the 2^-ΔCt^ method (41). The Shapiro-Wilk test was conducted to assess the normality of the data. Statistical analysis included the Kruskal-Wallis test to assess overall differences across groups and the Mann-Whitney U test with P values adjusted by Bonferroni correction to evaluate specific pairwise comparisons.

## 3 Results

### 3.1 The immune gene expression landscape

**Figure 2:**
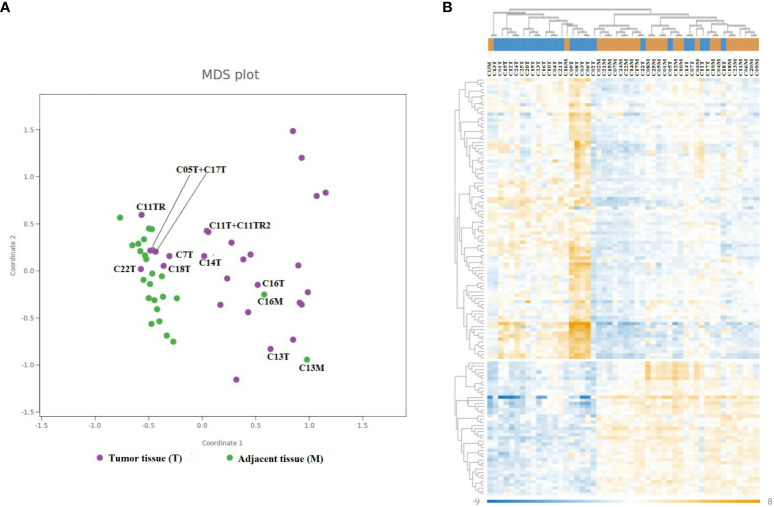
Differential gene expression of immune-related genes in CRC. The MDS plot **(A)** and heatmap **(B)** illustrate similarities in the mRNA expression of 579 immune-related genes in 25 cancer samples and 25 paired adjacent non-neoplastic tissue samples. **(A)** Cancer samples are depicted in violet, and adjacent non-neoplastic samples are depicted in green. Selected samples are annotated with sample ID (number) and type of tissue (T, cancer; M, adjacent non-neoplastic tissue). **(B)** The columns represent the samples, where cancer samples are colored blue and non-neoplastic samples are colored orange in the dendrogram on top. The rows represent individual genes. The color scale indicates the relative expression levels of each gene, where dark blue represents low expression and dark orange represents high expression.

In our study, we analyzed colorectal tumors and adjacent non-neoplastic tissues from 25 patients using the nCounter Immunology V2 panel to assess the expression of 579 immune-related genes. Our methodology involved normalizing the expression data and employing multidimensional scaling (MDS) for visualization, revealing diverse expression patterns across the samples. Notably, tumor samples exhibited a scattered distribution in the MDS plot (**Figure 2A**), indicative of their heterogeneous immune gene expression. This contrasted with the more uniform expression profiles seen in adjacent non-neoplastic tissues. A subgroup of tumors (C03T, C06T, C08T, and C09T) clustered apart from the other samples in the MDS plot, suggesting gene expression in this subgroup that differed from the other tumor samples. Further analysis revealed a similarity in immune gene expression between two non-neoplastic samples (C13M and C16M) and their corresponding tumor samples. This suggests similar immune gene expressions between the tumor sample and the paired adjacent non-neoplastic tissue sample and may indicate that the inflammatory response in the tumor of these patients extended to the surrounding adjacent tissue.

Additionally, our heatmap hierarchical clustering analysis corroborated the MDS findings, grouping the same four tumor samples together (C03T, C06T, C08T, and C09T), all of which displayed heightened expression of certain immune genes compared to adjacent non-neoplastic tissue (**Figure 2B**).

### 3.2 Differentially expressed genes (DEG) of the paired samples

**Figure 3:**
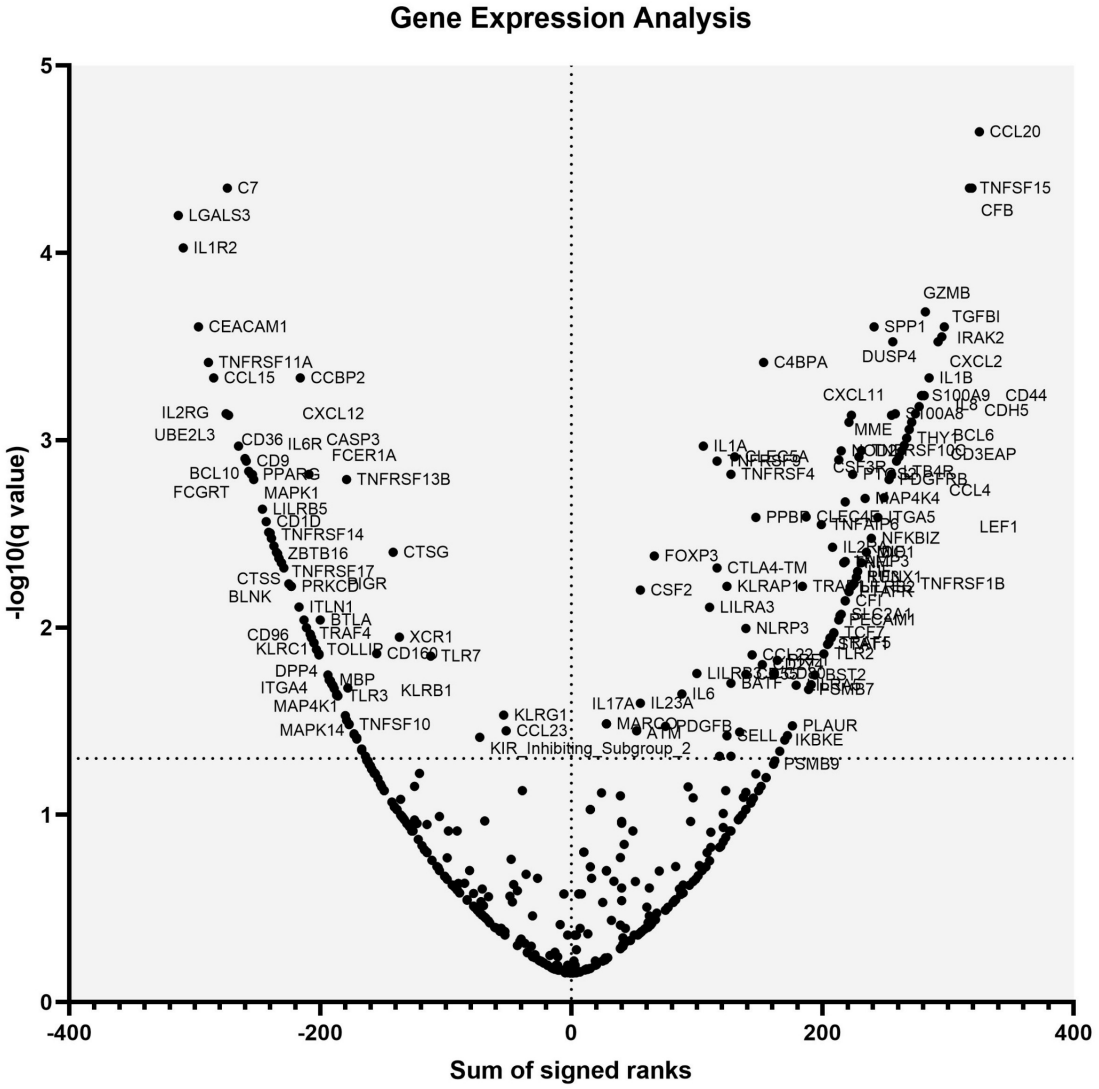
Comparison of mRNA expression of individual genes in paired cancer and adjacent non-neoplastic samples. The volcano plot shows the Wilcoxon test results, comparing mRNA expression in paired cancerous and non-neoplastic samples.

The Wilcoxon signed rank test was employed to analyze the differences in transcription levels between tumor biopsy and its adjacent non-tumor tissue, as illustrated in **Figure 3**. This analysis led to the identification of 202 differentially expressed genes. Among these, 120 DEGs were more highly expressed in tumor tissues, whereas 82 showed greater expression in adjacent non-neoplastic tissues. The genes exhibiting the highest levels of expression in tumor tissues included CCL20, CFB, TNFSF15, GZMB, TGFBI, SPP1, CXCL2, DUSP4, IRAK2, C4BPA, IL1B, CD44, CDH5, CXCL1, IL8, IL1RAP, LEF1, S100A9, and IFITM1. Conversely, genes such as C7, LGALS3, IL1R2, CEACAM1, TNFRSF11A, CCBP2, CCL15, CXCL12, IL2RG, UBE2L3, CD36, CASP3, BCL10, CD9, FCGRT, FCER1A, IL6R, PPARG, TNFRSF13B, and MAPK1 were found to have the lowest expression levels in the comparative analysis. Detailed information on all DEGs and their official full names is available in **Supplementary Table 1**.

### 3.3 Chemokines and chemokine receptors

Our analysis revealed that a significant overexpression of 120 genes was observed within the tumor samples. Subsequently, we identified genes consistently overexpressed across a broad spectrum of these samples. Notably, chemokine genes CXCL8, CXCL2, CXCL1, and CCL20 exhibited an average normalized count exceeding 2000 in the cancerous tissues (**Table 2**). In addition, CXCL8 exhibited a tenfold increase in expression in tumor samples compared to non-neoplastic counterparts in 76% of patients. Similar patterns of overexpression were observed for CXCL2, CXCL1, and CCL20, with increments of fivefold or more in 76%, 64%, and 44% of the cases, respectively. C-X-C Motif Chemokine Receptor 2 (CXCR2), a chemokine receptor with high affinity for CXCL8, was the only chemokine receptor identified as DEG in this study. The normalized counts in tumor tissue were low compared to several chemokines, and the counts in non-neoplastic tissue were nearly undetectable. 80% of the patients displayed 10-fold or more expression of CXCR2, suggesting infiltration of CXCR2-positive immune cells in most of the tumors.

**Table 2:** Chemokines and chemokine receptors overexpressed in cancer.

Chemokine/chemokine receptors		Adj. p value	Mean counts		Overexpression (%)					
			Tumor	Adjacent tissue	Tumor			Adjacent tissue		
					Folds					
					x10	x5	x3	x10	x5	x3
**CCL**	CCL20	<0.001	2140	609	28	44	56	0	0	0
	CCL2	0.0059	480	180	12	20	40	12	12	16
	CCL3	0.0012	381	20	48	48	48	4	12	12
	CCL4	0.0015	306	51	28	48	56	0	0	4
**CXCL**	CXCL8*	<0.001	19658	557	76	80	80	0	0	0
	CXCL1	<0.001	4596	593	44	64	80	0	0	0
	CXCL2	<0.001	2209	308	40	76	84	0	0	0
	CXCL10	0.0061	654	163	28	48	56	4	8	12
	CXCL7**	0.0026	590	4	60	64	68	0	4	4
	CXCL9	0.0202	376	162	32	40	52	8	16	16
	CXCL11	<0.001	276	33	56	68	72	0	0	0
**CXCR**	CXCR2	<0.001	193	14	80	80	80	0	0	0

*(IL8); **(PPBP).

### 3.4 Cytokines and cytokine-mediated signaling

Our study highlighted that the pro-inflammatory cytokine gene Interleukin-1β exhibited an average normalized transcript count of 1711 in tumor samples (**Table 3**). Moreover, over half (52%) of the patients had a tenfold increase in IL1B expression levels. IL1A, exhibiting similarities to IL1B, displayed a tenfold increase in 44% of the tumor samples, though its average normalized count in tumors was notably lower. The overexpression of IL1RAP, which encodes a part of the IL1 receptor heterodimer, underscores the activation of the IL1 signaling pathway within tumor environments. Additionally, the expression of IRAK2 and various members of the TNF family, including TNFα and Vascular Endothelial Growth Inhibitor, TNFSF15, was observed (**Table 3**). Significant upregulation of multiple cytokines recognized by type I cytokine receptors was noted (see **Table 3**). IL6 expression was elevated tenfold or more in 44% of patients, and increased expression levels of Leukemia Inhibitory Factor (LIF), Colony Stimulating Factor 2 (CSF2), and IL23A were also observed. Activation of Janus Kinases (JAK) and Signal Transducers and Activators of Transcription (STAT) follows the recognition of cytokines by type I cytokine receptors. Our findings showed moderate overexpression of STAT1 as well as suppressors of cytokine signaling (SOCS) 1 and 3, corroborating the activation of type I receptor signaling pathways in the tumor tissues.

**Table 3:** Cytokines and cytokine mediated signaling overexpressed in cancer.

Cytokine and cytokine signaling		Adj. p value	Mean counts		Overexpression (%)					
			Tumor	Adjacent tissue	Tumor			Adjacent tissue		
					Folds					
					x10	x5	x3	x10	x5	x3
**Il1 cytokine and signaling**	IL1B	<0.001	1711	92	52	64	72	0	0	0
	IL1A	0.001	120	1	44	52	56	0	0	0
	IRAK2	<0.001	132	34	20	36	44	0	0	0
	IL1RAP	<0.001	100	35	0	24	36	0	4	4
**TNF**	TNFSF15	<0.001	251	76	12	28	68	0	0	0
	TNF	0.005	70	20	40	52	60	0	4	8
	TNFSF11	0.049	41	15	32	40	52	8	8	16
	TRAF5	0.012	570	322	4	4	16	0	0	0
**Type I cytokine receptor signaling**	LIF	0.005	403	140	12	12	28	0	4	4
	IL6	0.023	290	12	44	44	52	0	4	4
	CSF2	0.006	47	2	16	24	36	0	0	0
	IL23A	0.025	25	2	32	40	40	4	4	4
	CSF3R	0.001	139	11	60	60	76	0	0	0
	IL2RA	0.004	58	19	52	56	56	4	4	8
	STAT1	0.012	949	653	0	8	12	0	0	0
	SOCS3	<0.001	832	146	24	52	68	0	4	4
	SOCS1	<0.001	131	44	28	32	48	4	4	4
**Platelet derived growth factor**	PDGFB	0.034	15	5	28	32	44	0	0	0
	PDGFRB	0.002	839	320	8	12	36	0	0	0
	MIF	0.004	2671	1636	4	4	8	0	0	4
	PTAFR	0.006	99	38	20	24	40	0	0	0

### 3.5 Host-pathogen interactions

We compared the expression of the genes associated with host-pathogen interactions (**Table 4**). Notably, genes S100A1 and S100A8, which encode for the calprotectin complex, demonstrated a significant upregulation, with a threefold increase in expression observed in 80% of the tumor samples. Among the Toll-like receptors, Toll-Like Receptor 2 (TLR2) was uniquely identified as being overexpressed in cancerous tissues. Furthermore, our observations revealed an upregulation of C-type lectin receptors, specifically C-Type Lectin Domain Family 5 Member A (CLEC5A) and C-Type Lectin Domain Family 4 Member E (CLEC4E). CLEC5A encodes the activating receptor Myeloid DAP12-associating lectin 1 receptor 1 (MDL-1), which recognizes several viral proteins. CLEC4E encodes the activating receptor Mincle, which exhibits an affinity for glycolipids like the mycobacterial cord factor (42). Elevated expression of these pathogen recognition receptors in tumor samples implies infiltration of myeloid cells within the TME.

**Table 4:** Genes related to host pathogen interactions overexpressed in cancer.

Host Pathogen Interaction		Adj. p-value	Mean counts		Overexpression (%)					
					Tumor			Adjacent tissue		
			Tumor	Adjacent tissue	Folds					
					x10	x5	x3	x10	x5	x3
**Extracellular**	S100A9	<0.001	1910	57	60	60	80	0	0	0
	S100A8	<0.001	909	1	80	80	84	0	0	4
**Surface receptors**	IFITM1	<0.001	13608	4306	16	32	48	0	0	0
	BST2	0.018	384	163	4	8	16	0	0	8
	FGR3A/B	<0.001	1021	277	16	48	60	0	0	0
	TLR2	0.014	128	30	32	36	40	8	8	8
	CLEC5A	0.001	123	3	44	52	56	0	0	0
	CLEC4E	0.003	79	6	56	60	68	0	0	8
**Intracellular receptors**	NOD2	0.001	66	18	28	44	44	0	0	0
	NLRP3	0.010	49	4	32	48	48	4	12	12

### 3.6 Cell adhesion receptors and extracellular proteins

The genes TGFBI, SPP1, and TNFAIP6 (Tumor Necrosis Factor Alpha-Induced Protein 6) encode secreted proteins integral to the extracellular matrix. Both TGFBI and SPP1 were observed to have high average normalized counts within tumor tissues (**Table 5**). Nevertheless, their expression in non-neoplastic samples exhibited variance; TGFBI maintained relatively high counts, while SPP1 was undetectable in half of the patient samples (**Table 5**; **Supplementary Table 1**). Several cell adhesion receptors, such as CD44 and PECAM1 (Platelet Endothelial Cell Adhesion Molecule 1), were highly expressed in tumor samples (**Table 5**). However, only a minority of patients showed expression levels of these genes at tenfold or fivefold in tumor tissues compared to their non-neoplastic counterparts, a finding attributed to significant expression in the adjacent mucosa as well (**Table 5**).

**Table 5:** Cell adhesion receptors and extracellular proteins overexpressed in CRC.

Cell adhesion and extracellular matrix		Adj. p value	Mean counts		Overexpression (%)					
			tumor	adjacent tissue	Tumor			Adjacent tissue		
					Folds					
					x10	x5	x3	x10	x5	x3
**Extracellular**	TGFBI	<0.001	5915	1971	12	28	52	0	0	4
	SPP1	<0.001	2810	48	64	76	76	4	4	4
	TNFAIP6	0.003	113	5	48	52	56	0	12	16
**Surface receptor**	CD44	<0.001	3543	1774	4	12	32	0	0	4
	PECAM1	0.009	1077	714	0	8	12	0	0	4
	ITGA5	0.003	928	218	16	28	44	4	4	4
	ICAM1	<0.001	569	147	16	16	44	4	4	4

### 3.7 Additional genes exhibiting overexpression in CRC tumors

Our investigation has further highlighted a series of genes significantly overexpressed within colorectal tumor tissues. As detailed in **Table 6**, these genes are characterized by at least a tenfold increase in expression in 40% or more of the tumors analyzed. Notably, granzyme B (GZMB) displayed a threefold change in expression in 76% of the tumor samples. GZMB is located in the granules of cytotoxic lymphocytes and plays a crucial role in inducing apoptosis in target cells following recognition. Furthermore, our analysis uncovered a high expression of PTGS2 (Prostaglandin-Endoperoxide Synthase 2), with a fivefold or greater increase observed in 68% of the tumor samples (**Table 6**). PTGS2 is responsible for the synthesis of cyclooxygenase 2, an enzyme that converts arachidonic acid into prostaglandins. Additionally, CD276, also known as B7-H3, a member of the B7 family of transmembrane surface molecules, demonstrated a significant expression, with a tenfold increase noted in 44% of the tumor samples. The elevated expression of CD276 suggests its potential as a target for checkpoint immunotherapy (43).

**Table 6:** Additional genes exhibiting overexpression in CRC.

Cellular function		Adj. p value	Mean counts		Overexpression (%)					
			Tumor	Adjacent tissue	Tumor			Adjacent tissue		
					Folds					
					x10	x5	x3	x10	x5	x3
**Apoptosis**	GZMB	<0.001	378	66	68	76	76	0	0	0
**Metabolism**	PTGS2 (COX-2)	0.002	618	55	60	68	72	0	4	8
**B7 family ligand**	CD276	0.005	1113	661	44	48	48	0	0	0

### 3.8 A subgroup of highly inflamed CRC tumors

**Figure 4:**
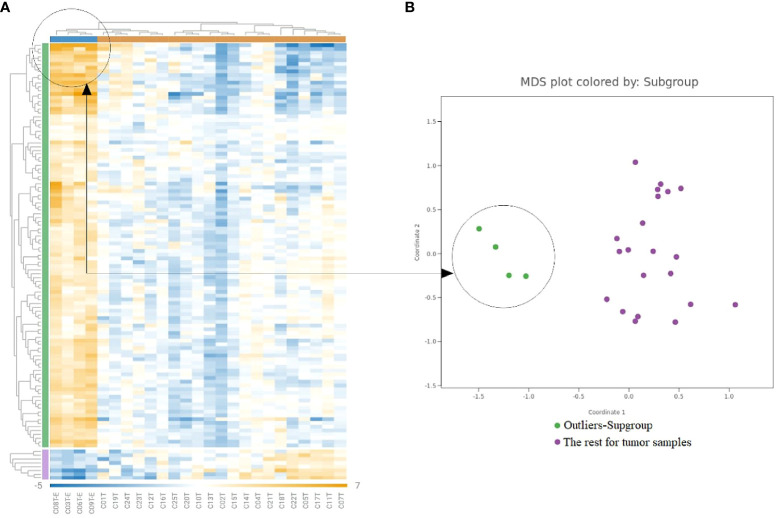
Gene expression analysis in the outliers-subgroups. **(A)** Heatmap clusters gene expression levels, highlighting a subgroup with increased immune gene expression. **(B)** The MDS plot differentiates the subgroup of highly inflamed tumors from ‘Regular’ samples, emphasizing their distinct expression patterns.

As shown in **Figure 2**, a subset of tumor samples from CRC patients, specifically C03T, C06T, C08T, and C09T, displayed notably enhanced expression of a range of genes in comparison to other tumor samples within the study. To delve deeper into the molecular distinctiveness of this subset, a differential gene expression analysis was carried out, contrasting these particular samples against the broader tumor sample collection (**Figure 4**). DEGs—65 out of 202 DEGs identified previously were significantly upregulated in this distinct group, as highlighted in **Supplementary Table 2**.

**Table 7** displays 19 genes exhibiting a greater than tenfold increase in expression, marking them as the most significantly upregulated genes in this subgroup. These genes encode proteins such as interleukins, chemokines, and other components of the inflammatory response. Notably, IL6 emerged as the gene with the highest upregulation, demonstrating a remarkable 103-fold increase in this group of cancers compared to the rest of the cancer samples. Similarly, SPP1, CXCL8, and C-C Motif Chemokine Ligand 3 (CCL3) also showed significant elevations in expression, with fold changes of 54, 34, and 34, respectively (**Table 7**). The pronounced fold changes in these genes, together with their adjusted p-values, emphasize the strength and significance of the differential expression within this highly inflamed subgroup. For further details, **Supplementary Table 1** enumerates the rest of the significantly upregulated genes that did not reach the tenfold change threshold, providing a broader perspective on the molecular alterations characterizing this subset.

**Table 7:** Genes highly expressed in a subgroup of CRCs.

Annotation	Gene	Adj. P-value	Mean		Fold* Change
			C03T, C06T, C08T, C09T	Remaining tumors	
**Chemokines**	CXCL8	<0.001	114909	3414	34
	CCL3	<0.001	2403	88	34
	CCL4	<0.001	1611	128	14
	CXCL11	<0.001	1549	108	17
**IL1 Cytokine receptor signaling.**	IL1B	<0.001	9867	357	29
	IL1A	<0.001	746	45	25
**Type I Cytokine receptor signaling**	SOCS3	<0.001	3560	374	10
	IL6	<0.001	1616	78	103
	CSF3R	<0.001	686	80	11
**Cell adhesion and extracellular matrix**	SPP1	<0.001	16601	323	54
	ICAM1	<0.001	2662	256	11
	TNFAIP6	<0.001	720	51	21
**Host-Pathogen interaction**	S100A8	<0.001	4407	329	14
	CLEC5A	<0.001	716	40	29
	TLR2	<0.001	616	80	10
	CLEC4E	<0.001	467	53	12
	NLRP3	<0.001	329	38	15
**Lymphocyte activation**	CD274	<0.001	385	46	13
**Metabolism**	IDO1	<0.001	2793	133	24

*Fold change of geometric mean.

### 3.9 Gene expression profiling in the cancer, polyp, and control groups

To identify potential biomarker genes for early detection of CRC, RT-qPCR analyses targeting a subset of genes were performed on all biopsy samples from cancer patients, adenoma patients, and controls, for a total of 228 biopsies. Target genes for RT-qPCR were selected based on nCounter data and the following criteria: (1) adjusted p-value Wilcoxon’s test below 0.05; (2) gene expression higher than 3-fold in tumor versus non-neoplastic tissue in a minimum of 70% of cancer patients; and (3) high average nCounter counts in tumor tissue (above 500). These selection criteria identified CXCL1, CXCL2, CXCL8, PTGS2, and SPP1 as candidate genes for further analysis. Additionally, C-X-C Motif Chemokine Ligand 9 (CXCL9) was included based on high nCounter counts in a small adenoma sample set (data not shown), and IL6 was included based on literature studies (44–46). Finally, TGFB1 was included as a control in our analysis due to consistently high expression levels observed across both tumor and adjacent non-neoplastic tissues in nCounter NanoString data (counts ranging from 340 to 430).

**Figure 5:**
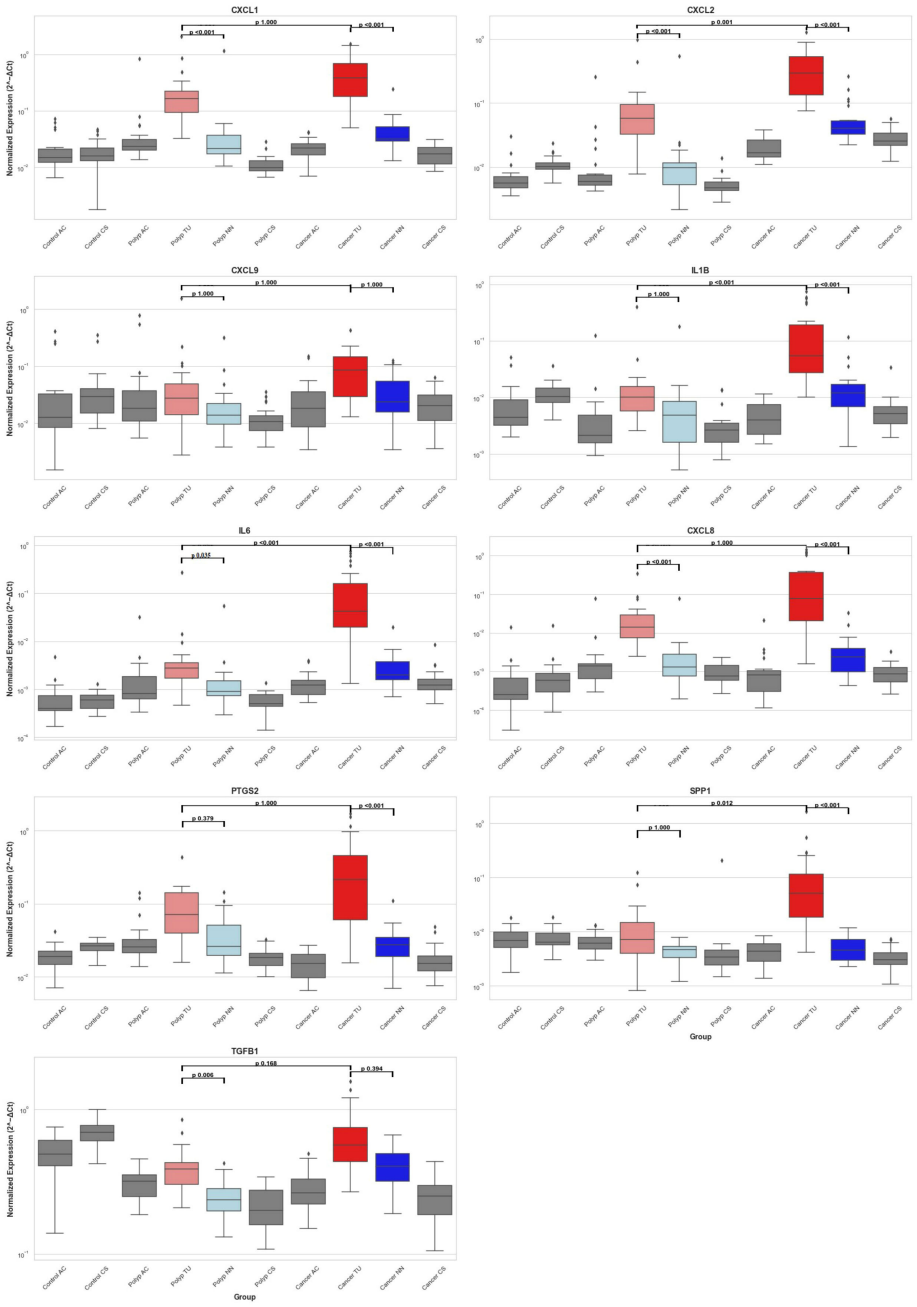
Distribution of Inflammatory Gene Expression Across Colorectal Tissues. This figure presents box plots showing the mRNA expression levels of nine genes (CXCL1, CXCL2, CXCL9, IL1B, IL6, CXCL8, PTGS2, SPP1, and TGFB1) measured by RT-qPCR and normalized against endogenous control genes GAPDH and POLR2A across different colorectal tissue samples. The samples include healthy controls from the ascending colon (Control AC) and sigmoid colon (Control CS), polyp patients with tissues from the ascending colon (Polyp AC), adenomas polyp (Polyp TU), adjacent non-neoplastic tissue (Polyp NN), and sigmoid colon (Polyp CS), and CRC patients with tissues from the ascending colon (Cancer AC), tumor (Cancer TU), adjacent non-neoplastic tissue (Cancer NN), and sigmoid colon (Cancer CS). Box plots indicate the median (line inside the box), interquartile range (box), and range (whiskers), with outliers shown as individual points outside the whiskers. The statistical significance of the comparisons is indicated within the plots with black lines: polyp TU vs. polyp NN, cancer TU vs. cancer NN, and polyp TU vs. cancer TU. P-values are displayed above the comparison lines.

The qPCR gene expression data are presented through boxplots (**Figure 5**) and **Table 8**, while detailed quantitative information is provided in **Supplementary Table 2**. We observed differential gene expression patterns across various tissue types, with a focus on polyp tissue (polyp TU) and tumor tissue (cancer TU), alongside adjacent tissue (NN) for context. The median expression levels of genes in cancer TU were predominantly higher than those in polyp TU, as is evident for particular genes (**Figure 5**). This indicates enhanced gene activity within the tumor environment. Cancer TU not only demonstrated elevated expression but also exhibited substantial variability across samples, signifying a degree of heterogeneity within tumor gene expression. Contrastingly, polyp TU generally showed lower and more stable expression levels, except for a few outliers. Although cancer adjacent tissue (NN) showed some level of expression for most genes, it was generally lower than those observed in tumor tissue but higher than in control. This pattern accentuates the complexity and dynamic nature of gene expression in the varying tissue states associated with CRC.

**Table 8:** Comparative RT-qPCR analysis of gene expression across different colon tissue.

Gene	RT-qPCR Results											
	Control (AC) vs. Control (CS)		Polyp (TU) vs. Control (CS)		Cancer (TU) vs. Control (CS)		Polyp (TU) vs. Polyp (NN)		Cancer (TU) vs. Polyp (TU)		Cancer (TU) vs. Cancer (NN)	
	FC	Adj. P-value	FC	Adj. p-value	FC	Adj. P-value	FC	Adj. P-value	FC	Adj. P-value	FC	Adj. P-value
**CXCL1**	1	1,000	18	<0,001	30	<0,001	11	<0,001	3	1,000	13	<0,001
**CXCL2**	2	0,144	11	<0,001	36	<0,001	12	<0,001	6	0,001	8	<0,001
**CXCL9**	3	1,000	4	1,000	3	1,000	8	1,000	3	1,000	4	1,000
**IL1B**	2	1,000	3	1,000	15	<0,001	9	1,000	15	<0,001	15	<0,001
**IL6**	1	1,000	25	<0,001	276	<0,001	13	0,035	51	<0,001	66	<0,001
**CXCL8**	4	1,000	56	<0,001	524	<0,001	22	<0,001	20	1,000	132	<0,001
**PTGS2**	1	1,000	4	0,003	16	<0,001	3	0,379	6	1,000	15	<0,001
**SPP1**	1	1,000	2	1,000	22	0,005	4	1,000	21	0,012	33	<0,001
**TGFB1**	1	0,255	1	0,002	1	1,000	2	0,006	2	0,168	2	0,394

AC, Ascending colon; CS, Sigmoid colon; TU, Neoplastic; NN, Adjacent non-neoplastic, FC, Fold Change.

To evaluate the differences in tumor and polyp tissue across the groups and within each group, we used the Kruskal-Wallis rank sum test. There was a statistically significant difference between the groups for all genes in both tumors and polyps (Kruskal-Wallis, p = <0.001), except for CXCL9 (Kruskal-Wallis, p = 0.05) (**Supplementary 2**). Next, we performed group comparisons using the Mann-Whitney U test and corrected the P values using the Bonferroni correction (**Table 8**). Our results suggest that there was no statistically significant variation in gene expression between biopsies from two different colon locations in the control group [Control (AC) and Control (CS)]. There was no significant difference in the expression of CXCL9 among the groups, as determined by the Kruskal-Wallis rank sum test. The immune genes CXCL1, CXCL2, IL1B, IL6, CXCL8, PTGS2, and SPP1 demonstrated elevated expression levels in tumor tissue (TU) relative to all other tissues, thus validating the results obtained from the NanoString analysis (**Supplementary 1**). CXCL1, CXCL2, IL6, and CXCL8 exhibited higher expression levels in polyps (TU) when compared to both control tissue (CS) and healthy tissues adjacent to the polyps (NN). In contrast, PTGS2 showed elevated expression exclusively in polyps (TU) when compared to the control group (CS) (**Table 8**). The comparison of tumor tissue (TU) and adenomatous polyp tissue (TU) highlights that the expression of CXCL1, CXCL8, and PTGS2 does not differ significantly (adj. p value = 1). This suggests that these genes are equally overexpressed in both cancers and polyps (**Table 8**).

## 4 Discussion

During the transformation of normal colonic epithelial tissues through adenomatous stages to carcinoma, the immune system emerges as a critical player in the progression of CRC (6, 47, 48). This research was designed to elucidate the immunological gene signatures present in colorectal tumors, with a particular focus on identifying genes that can differentiate between adenomatous and carcinomatous stages in a Norwegian cohort. Through the comprehensive analysis of 579 genes implicated in the inflammatory and immune response, our study identified a significant elevation in the expression of 120 genes within tumor samples. These genes underwent further evaluation to ascertain average expression levels and the frequency of enhanced expression in tumors compared to adjacent non-cancerous tissues. This process led to the selection of eight pivotal immune-related genes for an in-depth investigation in a larger sample group comprising cancer patients, individuals with adenomatous polyps, and healthy controls. The analysis revealed patient-specific immune profiles, pointing to the CRC tumor microenvironment’s diversity, which is composed of cancer cells, immune cells, stromal cells, and various signaling molecules (6, 8, 49). Interestingly, four tumor samples demonstrated exceptionally high expression levels of several genes, distinguishing them from the rest and underscoring the immune system’s heterogeneity within CRC contexts, as illustrated in **Figure 4**. This variation in gene expression not only emphasizes the intricate interaction between the TME and the immune system but also proposes these genes as potential biomarker candidates for patient stratification.

Increased expression of CXCL-type chemokines, notably CXCL1, CXCL2, and CXCL8, was observed in colorectal tumor tissues. These chemokines serve as potent neutrophil attractants, facilitating their recruitment to the TME (50). Although neutrophils are known to possess anti-tumor immunity, in the context of cancer, they often promote tumor growth and metastasis (51–53). Our results also demonstrated increased expression of the CXCR2 receptor in cancer. CXCR2 is a G protein-coupled receptor mainly expressed on neutrophils and other myeloid cells that specifically binds to several CXCL chemokines, including CXCL1, CXCL2, and CXCL8. The CXCL8/CXCR2 pathway is also involved in mediating the movement of M2 macrophages in pancreatic cancer, and a study by Shao et al. showed that CXCL8 promotes M2 macrophage polarization and hinders CD8+ T cell infiltration, leading to the development of an immunosuppressive microenvironment in CRC (54, 55). The expression levels of CXCL1, CXCL2, and CXCL8 were significantly elevated, showing a more than threefold increase in over 80% of the analyzed tumor samples. Notably, CXCL8 was found to be 34 times more prevalent in the CRC subgroup (as indicated in **Table 7**). Additionally, expression of these genes was also increased in adjacent non-neoplastic tissue compared to the other colon locations in cancer patients. This suggests that the surrounding tumor tissue is also inflamed. Furthermore, CXCL1 and CXCL8 expression in adenomatous polyps reached levels comparable to those observed in tumor tissues (**Figure 5**) (56, 57). That suggests that these chemokines are involved in the initial stages of epithelial transformation, highlighting inflammation as a probable component of polyp lesions (28). The correlation between increased CXCL1 expression and cancer progression, invasion, and adverse patient outcomes has led to its proposal as a non-invasive biomarker for CRC (58). However, the findings of this study suggest that while this chemokine may not serve as a specific marker for cancer, it could potentially function as an indicator for the presence of adenomatous polyps and CRC.

In CRC patients, cytokines and cytokine receptors such as IL1B, a pro-inflammatory cytokine, exhibited the highest mean expression levels among tumor-associated cytokines, with 72% of patients showing more than a threefold increase in tumor tissues compared to adjacent non-neoplastic tissues (**Table 3**), and a 29-fold increase observed in a specific tumor subgroup (**Table 7**). A particular subtype of cancer-associated fibroblasts (CAFs) characterized by elevated IL-1 signaling, termed IL1R1+, has been identified in CRC, which facilitates tumor growth and immune suppression, underscoring the profound influence of IL-1 pathways on the TME by modulating tumor proliferation and immune response mechanisms (59). Our study found that, compared to adenomatous polyps where IL1B expression levels were unchanged, a notable increase in expression was evident in the transition phase toward carcinoma (**Figure 5**), suggesting its potential utility as a non-invasive biomarker for CRC. This is supported by Krzystek-Korpacka et al. research, which found elevated circulating IL1B levels in cancer patients but not in those with adenomas, aligning with our observations that IL1B may differentiate cancer from adenomatous conditions. However, IL1B levels are also significantly elevated in patients with active inflammatory bowel disease (IBD) (23), indicating that IL1B should be considered alongside other biomarkers for accurate differentiation of intestinal diseases. The emerging cytokine networks in CRC underscore the critical role of cytokine signaling in the disease’s pathogenesis. Cytokines, including IL1B and other IL-1 family members, are associated with various aspects of CRC progression, promoting tumor immune evasion, cell survival, and proliferation (22, 60). The IL1 receptor accessory protein (IL1RAP), essential for signal transduction in IL1R, IL33R, and IL36R through dimerization and intracellular signaling initiation upon cytokine binding (61), was also found to be overexpressed in our study. The observed overexpression of IL1RAP and the substantial but similar expression levels of IL1R1 in tumor and adjacent non-neoplastic tissues further corroborate the significant role of IL-1 signaling in CRC. Our study suggests a significant role of IL-6 in the progression of CRC in the sense that there was significantly higher expression in cancerous tumors compared to adenomatous polyps. In addition, there was a notable 103-fold increase in the outlier CRC subgroup (**Table 7**), suggesting its potential as a biomarker for patient stratification (62). Signaling by the IL-6/JAK/STAT3 axis is prominent in many human cancers and regulates various cellular functions relevant to cancer advancement, such as inflammation, cell survival, and proliferation. We did not observe any significant difference in STAT3 expression and less expression of IL6R and IL6ST in cancer samples compared to paired non-neoplastic samples. However, we observed a relatively high transcript count for these proteins in both cancer samples and non-neoplastic tissue, suggesting the presence of cells capable of IL-6-mediated signaling in both healthy and pathologic mucosa. We found a significantly higher expression of STAT1 in cancer; however, the counts in non-neoplastic tissue were relatively high, and with 12% of the patients having threefold expression of STAT3 in cancer, the difference between cancer and non-neoplastic tissue was modest. In comparison, the expression of suppressors of cytokine signaling (SOCS), especially SOCS3, was overexpressed in cancer, suggesting the presence of a regulatory feedback system that controls JAK/STAT signaling in the investigated cancers (63).

S100 Calcium Binding Protein, S100A9, and S100A8 showed more than a threefold increase in expression in over 80% of colorectal tumor tissue samples in our study (64, 65). These proteins are believed to function as alarmins or damage-associated molecular patterns (DAMPs), released in response to cellular distress or injury. S100A8/A9 is believed to interact with pattern recognition receptors (PRRs) like Toll-like receptor 4 (TLR4) and the receptor for advanced glycation end products (RAGE), which triggers signaling pathways leading to the production of pro-inflammatory cytokines and chemokines, enhancing the immune response to infectious agents (66). The S100A8/A9 complex, known as calprotectin, is used as a biomarker for IBD, including Crohn’s disease and ulcerative colitis (67).

We identified heightened expression of TLR2 as well as CLEC5A and CLEC4E in cancer samples in general (**Table 4**) and in the subgroup (**Table 7**). The counts in cancer were relatively low, suggesting that the expression is restricted to immune cell infiltrations. Studies have shown that CLEC5A is positively related to immune infiltration, including macrophages, cancer-associated fibroblasts, and regulatory T cells (68, 69). Furthermore, CLEC5A expression correlates with several critical aspects of cancer biology, such as epithelial-mesenchymal transition (EMT) and apoptosis processes. It has been identified as a potential prognostic biomarker for diverse cancers and a target for anti-tumor therapy (68). Research on TLR2 expression in CRC is burgeoning, underscoring its crucial impact on tumor progression, the immune response, and patient outcomes. This body of work is enriching our comprehension of the intricate relationship between the immune system and the oncogenic process in CRC (70, 71). Given that the expression levels of host-pathogen recognition receptor genes in this study were relatively subdued compared to those of chemokines and cytokines, their utility as non-invasive biomarkers appears limited, leading to a decision against their further exploration in adenomatous polyps and control subjects. However, this insight still enhances our grasp of the immunological nuances present in colorectal tumors.

SPP1, also known as osteopontin (OPN), and its interactions with key cell adhesion molecules, including CD44 and ITGA5, showed significant elevations in our study. (**Table 5**). Notably, SPP1 showed more than a threefold increase in 76% of CRC samples. Pivotal roles of OPN in disease progression through cell adhesion, migration, and immune regulation have been suggested (72, 73). Research, such as the study by Kazakova et al., underscores SPP1’s involvement in angiogenesis within the CRC tumor microenvironment, particularly in how SPP1 expression correlates with the mobilization of tumor-associated macrophages and angiogenesis, critical for tumor growth and metastasis (74). These findings suggest SPP1’s potential as a prognostic biomarker for adverse outcomes in colon and rectal cancer (75, 76). Our research contributes to the increasing evidence linking SPP1 and its receptors, CD44 and ITGA5, to the development of CRC. Interestingly, SPP1 elevation in CRC, but not in adenomatous polyps, suggests its biomarker potential for cancerous developments rather than early-stage lesions.

PTGS2 (COX-2) has a role in various physiological processes, including its abnormal increase in cancer tissue, promoting tumor growth, angiogenesis, and metastasis (77). We observed a threefold increase in COX-2 expression in 72% of CRC tissues, suggesting its potential as a diagnostic biomarker for CRC. This is supported by the correlation found by Hamaya et al. between fecal COX-2 mRNA levels and CRC presence, indicating its non-invasive biomarker potential for CRC detection (78).

CD276, known for its dual role in the immune system (79), was found in our study to be highly expressed in tumor samples compared to adjacent non-neoplastic tissue (**Table 6**). Its heightened presence and role in modulating immune responses highlight CD276 as a promising target for immunotherapy (43, 80). Current research is exploring therapeutic strategies targeting CD276 and its pathways to enhance anti-tumor immune responses, potentially improving the efficacy of existing treatments or leading to new interventions for CRC patients (81).

## 5 Study limitations

The study was designed to initially identify genes that were highly expressed in tumors compared to non-neoplastic tissue and to investigate these genes further in other patient groups with the aim of identifying potential biomarkers. A limitation of this approach is the loss of potential gene candidates that are highly expressed in both tissues.

## 6 Conclusion

Our research on the immunological traits of CRC in the Norwegian cohort identified 202 genes with significant differential expression, including 120 upregulated in tumors and 82 in healthy tissues. We focused on eight genes (CXCL1, CXCL2, CXCL9, IL1B, IL6, CXCL8, PTGS2, and SPP1) due to their high expression levels, suggesting their utility as non-invasive biomarkers. Analyzing a variety of samples—CRC, adjacent non-neoplastic tissue, polyps, and controls across different colon sections—revealed that IL1B, IL6, and SPP1 are specifically overexpressed in CRC, distinguishing them from benign polyps. Similarly, CXCL1, CXCL2, CXCL8, and PTGS2 are upregulated in CRC and adenomatous polyps but not in normal tissue, highlighting their potential for CRC detection. While this study is descriptive, it establishes a foundational understanding of the immune landscape in CRC and adenomatous polyps. The differential expression patterns of these genes suggest their involvement in early carcinogenesis, thereby supporting their potential as biomarkers for early CRC detection. However, it should be noted that the expression of several of these markers may prolong and increase as CRC tumors advance to later stages (24). Future studies should aim to include larger patient cohorts to validate these findings and explore the functional roles of CXCL1, CXCL2, IL1B, IL6, CXCL8, PTGS2, and SPP1. Additional research is necessary to assess their efficacy in non-invasive screening methods, particularly in clinical settings.

## Data availability statement

The original contributions presented in the study are included in the article/**Supplementary Material**. Further inquiries can be directed to the corresponding author.

## Ethics statement

The study has been approved by both the regional committee for medical and health-related research ethics and the data protection manager at Ahus (REK 2012/1944). Patients were informed that further samples would be acquired prior to the procedure, and they were given the choice to abandon the study at any time. All participants involved gave written, informed consent. The studies were conducted in accordance with the local legislation and institutional requirements. The participants provided their written informed consent to participate in this study.

## Author contributions

TO: Conceptualization, Data curation, Formal Analysis, Investigation, Methodology, Project administration, Software, Validation, Visualization, Writing – original draft, Writing – review & editing. HT: Investigation, Methodology, Project administration, Supervision, Validation, Writing – review & editing, Resources, Conceptualization, Funding acquisition. DJ: Writing – review & editing, Data curation, Software, Visualization. SB: Writing – review & editing, Resources, Project administration. VB: Resources, Writing – review & editing, Investigation, Methodology, Project administration, Supervision, Validation, Conceptualization, Funding acquisition. PS: Conceptualization, Data curation, Formal Analysis, Investigation, Methodology, Supervision, Validation, Visualization, Writing – review & editing, Project administration.
